# Novel Immune-Related Gene Signature for Risk Stratification and Prognosis of Survival in ER (+) and/or PR (+) and HER2 (−) Breast Cancer

**DOI:** 10.3389/fphar.2022.820437

**Published:** 2022-06-02

**Authors:** Feng Du, Fangchao Zheng, Ying Han, Jiuda Zhao, Peng Yuan

**Affiliations:** ^1^ Key Laboratory of Carcinogenesis and Translational Research (Ministry of Education/Beijing), The VIPII Gastrointestinal Cancer Division of Medical Department, Peking University Cancer Hospital and Institute, Beijing, China; ^2^ Department of Medical Oncology, National Cancer Centre/National Clinical Research Center for Cancer/Cancer Hospital, Chinese Academy of Medical Sciences and Peking Union Medical College, Beijing, China; ^3^ Key Laboratory of Carcinogenesis and Translational Research (Ministry of Education /Beijing), Department of Palliative Care, Peking University Cancer Hospital and Institute, Beijing, China; ^4^ Breast Disease Diagnosis and Treatment Center, Affiliated Hospital of Qinghai University and Affiliated Cancer Hospital of Qinghai University, Xining, China; ^5^ Department of VIP Medical Services, National Cancer Centre/National Clinical Research Center for Cancer/Cancer Hospital, Chinese Academy of Medical Sciences and Peking Union Medical College, Beijing, China

**Keywords:** breast cancer, immune gene signature, prognosis, immune cell, cancer phenotypes

## Abstract

**Background:** Although intrinsic molecular subtype has been widely used, there remains great clinical heterogeneity of prognosis in the estrogen receptor (ER)- and/or progesterone receptor (PR)-positive and human epidermal growth factor receptor 2 (HER2)-negative breast cancer (BC).

**Methods:** The transcriptome expression data of messenger RNA (mRNA) were downloaded from The Cancer Genome Atlas (TCGA), Molecular Taxonomy of Breast Cancer International Consortium (METABRIC), and the Gene Expression Omnibus (GEO) databases. Immune-related genes were acquired from the ImmPort database and additional literature search. Univariate Cox, LASSO regression, and multivariate Cox regression were used to screen prognostic immune-related genes and establish the risk signature. The correlation between the risk signature and clinical characteristics, the abundances of immune cells within the tumor microenvironment, and cancer phenotypes were further assessed.

**Results:** Of note, 102 immune-related prognostic genes were identified in the METABRIC dataset by univariate Cox analysis. Consecutively, 7 immune-related genes (SHMT2, AGA, COL17A1, FLT3, SLC7A2, ATP6AP1, and CCL19) were selected to establish the risk signature by LASSO regression and multivariate Cox analysis. Its performance was further verified in TCGA and GSE21653 datasets. Multivariate Cox analysis showed that the risk signature was an independent prognostic factor. The 7-gene signature showed a significant correlation with intrinsic molecular subtypes and 70-gene signature. Furthermore, the CD4^+^ memory T cells were significantly higher in the low-risk group while a significantly higher proportion of M0-type macrophages was found in the high-risk group in both METABRIC and TCGA cohorts, which may have an influence on the prognosis. Furthermore, we found that the low-risk group may be associated with the immune-related pathway and the high-risk group was with the cell cycle-related pathway, which also showed an impact on the prognosis.

**Conclusion:** These seven immune-related gene risk signatures provided an effective method for prognostic stratification in ER (+) and/or PR (+) and HER2 (−) BC.

## Highlights

1. Based on TCGA, METABRIC, and GSE21653 cohorts, we analyzed the characteristics of TME in ER (+) and/or PR (+) and HER2 (−) breast cancer.
2. Seven immune-related genes were selected to establish the gene risk signature.
3. Gene risk signatures provided an effective method for prognostic stratification in ER (+) and/or PR (+) and HER2 (−) breast cancer.

## Background

Breast cancer (BC) ranks the first in female malignant tumors and poses a notable threat to women’s health worldwide. BC is a heterogeneous disease, demonstrating substantial intrinsic heterogeneity in terms of genetic, epigenetic, and phenotypic modifications and metabolism and is also affected by the estrogen receptor (ER) status, progesterone receptor (PR) status, and human epidermal growth factor receptor 2 (HER2) status (Baliu-Piqué et al., 2020). The ER (+) and/or PR (+) and HER2 (−) groups account for two-thirds of all BC (Ignatiadis and Sotiriou, 2013), which is sensitive to endocrine therapy and has a better prognosis than HER2-positive or triple-negative BC (TNBC). Although treated as the whole population, significant heterogeneity still exists in the ER (+) and/or PR (+) and HER2 (−) breast cancer (Cancer Genome Atlas Network, 2012; Curtis et al., 2012). This population has been shown to have different gene expression profiles, prognostic features, and sensitivity to endocrine therapy (Sørlie et al., 2001).

Currently, the most common method used for the classification of this population was according to the St Gallen criteria, which divided these patients into luminal-A and luminal-B subgroups by the immunohistochemical expression of ER, PR, and Ki67. However, some studies have reported that the luminal A/B classification does not fully distinguish the heterogeneity in ER (+) and/or PR (+) and HER2 (−) breast cancer (Gatza et al., 2014; Netanely et al., 2016; Zhu et al., 2019). Moreover, the optimal cut-off values of PR and ki67 to define the luminal-B subtype are still controversial (Focke et al., 2017). Consequently, novel biomarkers are needed for the effective discrimination of the ER (+) and/or PR (+) and HER2 (−) breast cancer.

The tumor microenvironment (TME) has been identified playing a critical role in tumor heterogeneity, but the mechanism has not been fully elucidated. Emerging evidence demonstrated that the TME comprises endothelial cells, fibroblasts, immune cells, and extracellular components that contribute to tumor heterogeneity (Jiang et al., 2021; Yuan et al., 2021). According to the characteristics of TME, BC was classified into different TME clusters (Xiao et al., 2019). The different TME clusters played an important role in tumor biology behavior and had an association with prognosis and even led to drugs that target TME specifically (Bareche et al., 2020).

Several prognostic risk tools were also used to clarify the association between TME with outcomes in tumors, including 70-gene prognostic risk score, OncotypeDX, and FoundationOne CDx. In patients with multiple myeloma, the results of a 70-gene prognostic risk score identified five main microenvironment clusters, and the clusters can be used to refine risk stratification to elucidate tumor treatment response. The specific cluster 5 with “low-granulocyte” was associated with poor survival (Danziger et al., 2020). A previous study by 21-gene recurrence score (OncotypeDX) showed that F/B from the tumor–stroma interface can be an independent prognostic indicator of metastasis-free survival in TME (Desa et al., 2020). Based on the 29 eligible patients and 523 patients/samples of TCGA database with metastatic head and neck squamous cell carcinoma, the phase II ALPHA study by FoundationOne CDx panel demonstrated that patients with MTAP mutation or loss led to a low fraction of CD8^+^ T cells and was associated with suppressed immune reaction factors in the TME (Kao et al., 2022).

Nonetheless, most BC molecular subtypes are mainly classified according to phenotypic characteristics, ignoring the supportive role of TME. Thus, it is necessary to explore new subtyping to improve the prognosis of this population. Here, our study aimed to discover key biomarkers in the TME of ER (+) and/or PR (+) and HER2 (−) breast cancer.

## Methods

### Data Acquisition and Preprocessing

Molecular Taxonomy of Breast Cancer International Consortium (METABRIC) and The Cancer Genome Atlas (TCGA) data were downloaded from the UCSC Cancer Browser website together with accompanying clinical information. The downloaded RNA-seq gene expression data were produced by the Illumina HiSeq platform and then RSEM-normalized and log2-transformed.

The GSE21653 cohort was derived from a study of gene expression profiling conducted on fresh frozen BC tissue collected from 266 patients in conjunction with thoroughly documented clinical data. All clinical and microarray data of these patients can be publicly downloaded at the GEO website (https://www.ncbi.nlm.nih.gov/geo/query/acc.cgi?acc=GSE21653).

The inclusion criteria included female; breast carcinoma with HER2 status negative and ER or PR status positive; complete clinical and follow-up information; and available RNA expression profile. The exclusion criteria included HER2 status positive or undetermined; both the ER and PR status negative or undetermined; incomplete follow-up information; and RNA expression data cannot be obtained. Finally, a total of 1,369 patients from the METABRIC dataset, 561 patients from TCGA dataset, and 129 patients from the GSE21653 dataset were enrolled.

### Bioinformatic Analysis

The R package “genefu” was used to classify the PAM50 molecular subtypes and calculate the 70-gene signature score of each case based on the gene expression data (Parker et al., 2009). To determine the optimal group number, we utilized the Nbclust and ConsensusClusterPlus R packages to perform the analysis (Marcum and Butts, 2015). The number of clusters tested by NbClust ranged between 2 and 10 clusters, and it can obtain the highest clustering number according to the calculation results. The parameters for the Nbclust algorithm were set as follows: Euclidean distance, K-means clustering, and index of all long. The ConsensusClusterPlus was performed based on the parameters including Euclidean distance, km clustering algorithm with 50 replicates and kmax of 10, pItem = 0.8, and pFeature = 0.8. The deconvolution approach CIBERSORT algorithm was performed on transcriptional expression data to estimate the proportions of twenty-two types of immune cells in each case using the CIBERSORT R package (Newman et al., 2015). The stromal, immune, and ESTIMATE scores of each sample were obtained from the website (Verhaak Lab; https://bioinformatics.mdanderson.org/estimate) (Yoshihara et al., 2013), which were used to characterize immune cell composition and calculate the ratio of immune-stromal components in the tumor microenvironment in a given sample.

We used the “limma” R package to identify the significantly differentially expressed genes (DEGs) between the high-risk and low-risk groups in TCGA dataset with the false discovery rate (FDR)-corrected *p*-value below 0.05 (Ritchie et al., 2015). The heatmap of the representative DEGs was generated using the package ComplexHeatmap in R version 3.6.1 (Gu et al., 2016). To explore different DEG-enriched signaling pathways in high- versus low-risk groups, the gene set enrichment analysis (GSEA) from those DEGs was conducted by the R package clusterProfiler using gene sets from the comprehensive Molecular Signatures Database (MSigDB) collections (Yu et al., 2012). Single-sample GSEA was performed using the GSVA Bioconductor package (Hänzelmann et al., 2013). We selected gene sets for various cell cycle- and immune-related pathways. For each case, the enrichment score of the selected gene set was obtained using the gene expression profile.

### Statistical Analyses

Statistical analyses were performed using SPSS version 23.0 (IBM, United States), GraphPad Prism version 8.00 (GraphPad Software, United States), and R version 3.6.1 (R Core Team, Vienna, Austria). Pearson’s chi-square test and Fisher’s exact test were used to compare the categorical variables and ordered categorical variables. Pearson correlation analysis was used to evaluate the association between two continuous variables. Mann–Whitney U tests were performed to evaluate the statistical significance within boxplots. Survival analysis was implemented by the log-rank test. LASSO Cox regression analysis (LASSO, least absolute shrinkage and selection operator) can achieve shrinkage and variable selection simultaneously by performing the Cox regression model with LASSO penalty. The LASSO Cox regression model was analyzed using the glmnet package. Univariate and multivariate regression analyses were performed with the Cox proportional hazards regression model to determine the parameters that were significantly correlated with prognosis. The statistical significance level with *p* value was set at 0.05.

## Results

### Identification of Prognostic Immune-Related Genes

**FIGURE 1:**
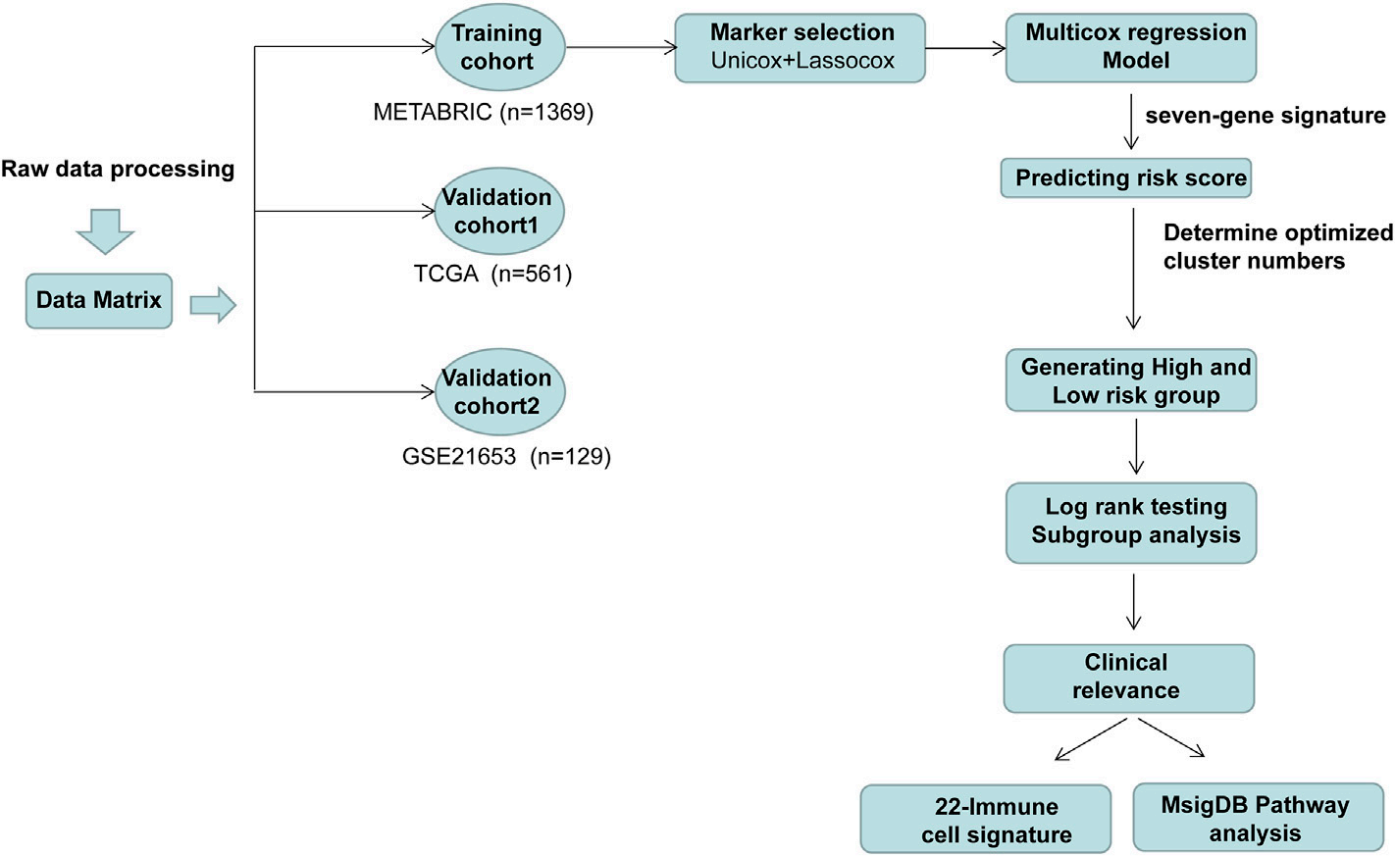
Flow chart showing the selection of immune-related genes and analysis process.

The workflow of this study is delineated in Figure 1. A total of 2,600 genes were identified from two datasets (CIBERSORT and ImmPort) (Bhattacharya et al., 2014; Newman et al., 2015) and literature search (Safonov et al., 2017; Xiao et al., 2019). Then, the gene expression profiles from 1,369 patients with ER (+) and/or PR (+) and HER2 (−) BC identified in the METABRIC dataset were used to perform the univariate Cox analysis. As a result, 102 genes were discovered to be significantly associated with overall survival.

**FIGURE 2:**
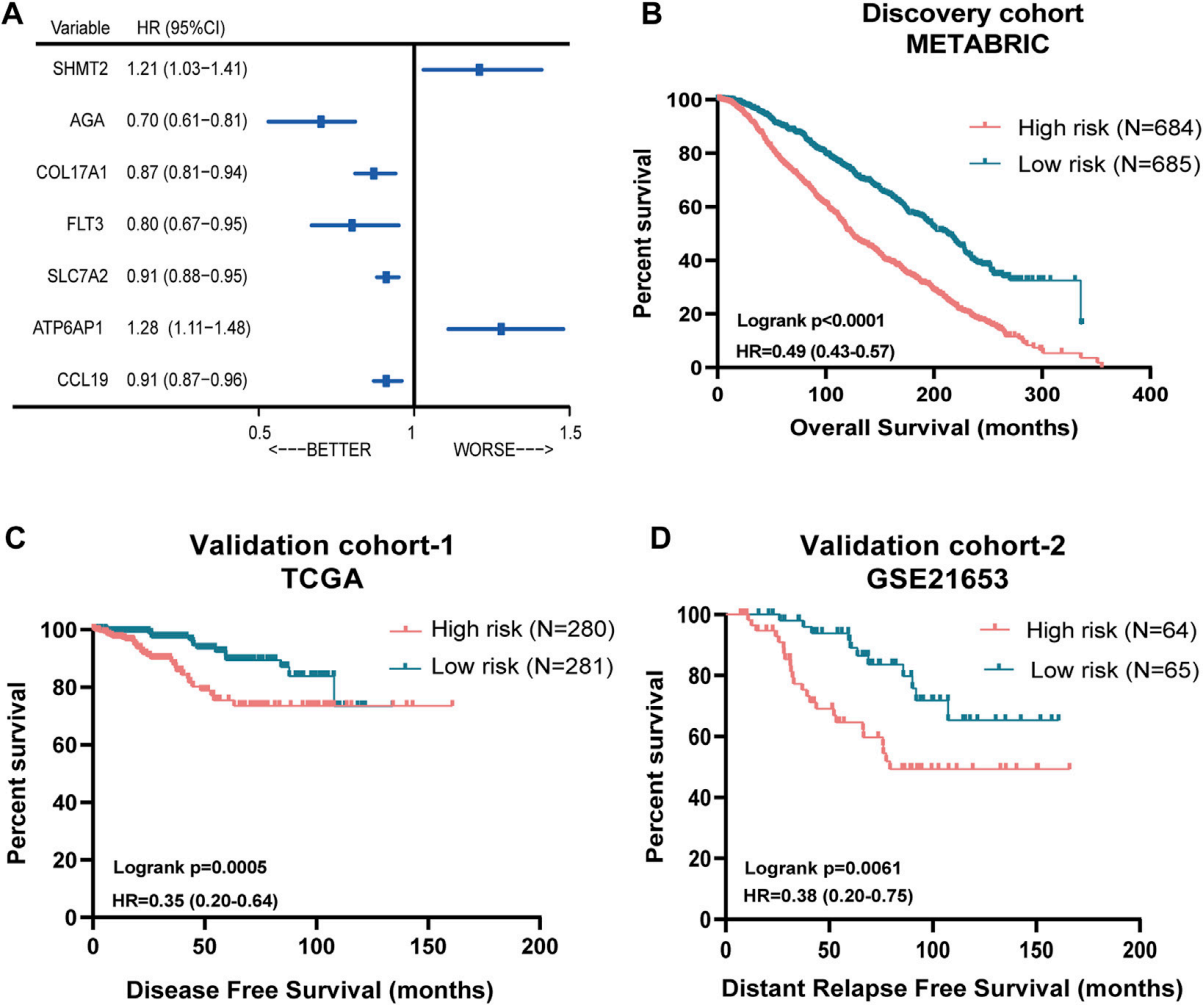
Prognostic significance of the seven-immune-related-gene IRS in ER (+) and/or PR (+) and HER2 (−) breast cancer. **(A)** Forest plot of prognostic significance of the seven immune-related genes. **(B)** Association of IRS with OS in the METABRIC cohort. **(C)** Association of RS with DFS in TCGA cohort. **(D)** Association of RS with OS in the GSE21653 cohort. IRS: immune risk signature; METABRIC: Molecular Taxonomy of Breast Cancer International Consortium; TCGA: The Cancer Genome Atlas.

Then, we performed the LASSO Cox regression analysis to eliminate the redundant collinearity and further validate the robustness (Supplementary Figures S1A, B). As a result, 7 genes were identified by LASSO regression analysis from the 102 genes. Then, we performed multivariate Cox regression analysis of the 7 genes in the METABRIC dataset, and ultimately, a prognostic signature comprising these 7 genes, including SHMT2, AGA, COL17A1, FLT3, SLC7A2, ATP6AP1, and CCL19, were selected to construct a prediction model. As shown in the forest plot, SHMT2 and ATP6AP1 were risk factors, whereas the other five genes were protective factors (Figure 2A). The comprehensive immune risk score (IRS) was imputed as follows: *IRS =* (0.19*SHMT2 value) *+* (−0.36*AGA value) *+* (−0.14*COL17A1 value) *+* (−0.23*FLT value) *+* (−0.09*SLC7A2) *+* (0.25*ATP6AP1) *+* (−0.09*CCL19).

The Nbclust and ConsensusClusterPlus analyses showed that the optimal number of clusters was two (Supplementary Figures S1C–E). Therefore, the median immune risk score was set as the cut-off value, and patients were categorized into two groups: high-risk (with higher IRS score) and low-risk group (with lower IRS score).

### Performance of the Immune Risk Score in ER (+) and/or PR (+) and HER2(−) Breast Cancer From METABRIC and TCGA Datasets

Figure 2B showed the survival discrimination power of IRS-based groups in the discovery cohort. Moreover, in order to further validate the prognostic predicting role of the IRS-based group, two different databases including TCGA and GSE21653 cohorts were included. As a result, patients in the low-risk groups showed consistently superior clinical outcomes than those in the high-risk groups in both datasets (Figures 2C,D).

Multivariate Cox analysis showed that the risk signature was the independent prognostic factor in both METABRIC and TCGA cohorts, after adjusting for established prognostic variables (Tables 1, 2).

**TABLE 1:** Multivariate Cox analysis of prognostic factors in the METABRIC cohort.

	*p*-value	HR	95% LI	95% UI
Tumor grade	0.753	1.02	0.88	1.19
TNM stage	<0.001	1.37	1.17	1.61
Risk group	<0.001	1.43	1.18	1.73
PAM50				
Basal	Reference	-	-	-
HER2	0.123	2.14	0.82	5.61
Luminal-A	0.089	2.18	0.89	5.36
Luminal-B	0.09	2.09	0.89	4.89
Normal	0.161	2.18	0.73	6.45
Number of positive node	<0.001	1.07	1.04	1.10
Age	<0.001	1.04	1.04	1.05
70-gene score	0.106	1.76	0.89	3.48

**TABLE 2:** Multivariate Cox analysis of prognostic factors in TCGA cohort.

	*p*-value	HR	95% LI	95% UI
Age	0.998	1.00	0.97	1.03
TNM stage	<0.001	2.86	1.83	4.48
PAM50				
Basal	Reference	-	-	-
HER2	0.978	0.00	0.00	
Luminal A	0.744	0.73	0.11	4.76
Luminal B	0.914	1.09	0.24	4.86
Normal	0.865	0.80	0.06	11.28
Risk group	0.002	3.25	1.54	6.90
70-gene score	0.631	0.57	0.06	5.77

### The Correlation Between the Immune Risk Score and Clinical Characteristics

**FIGURE 3:**
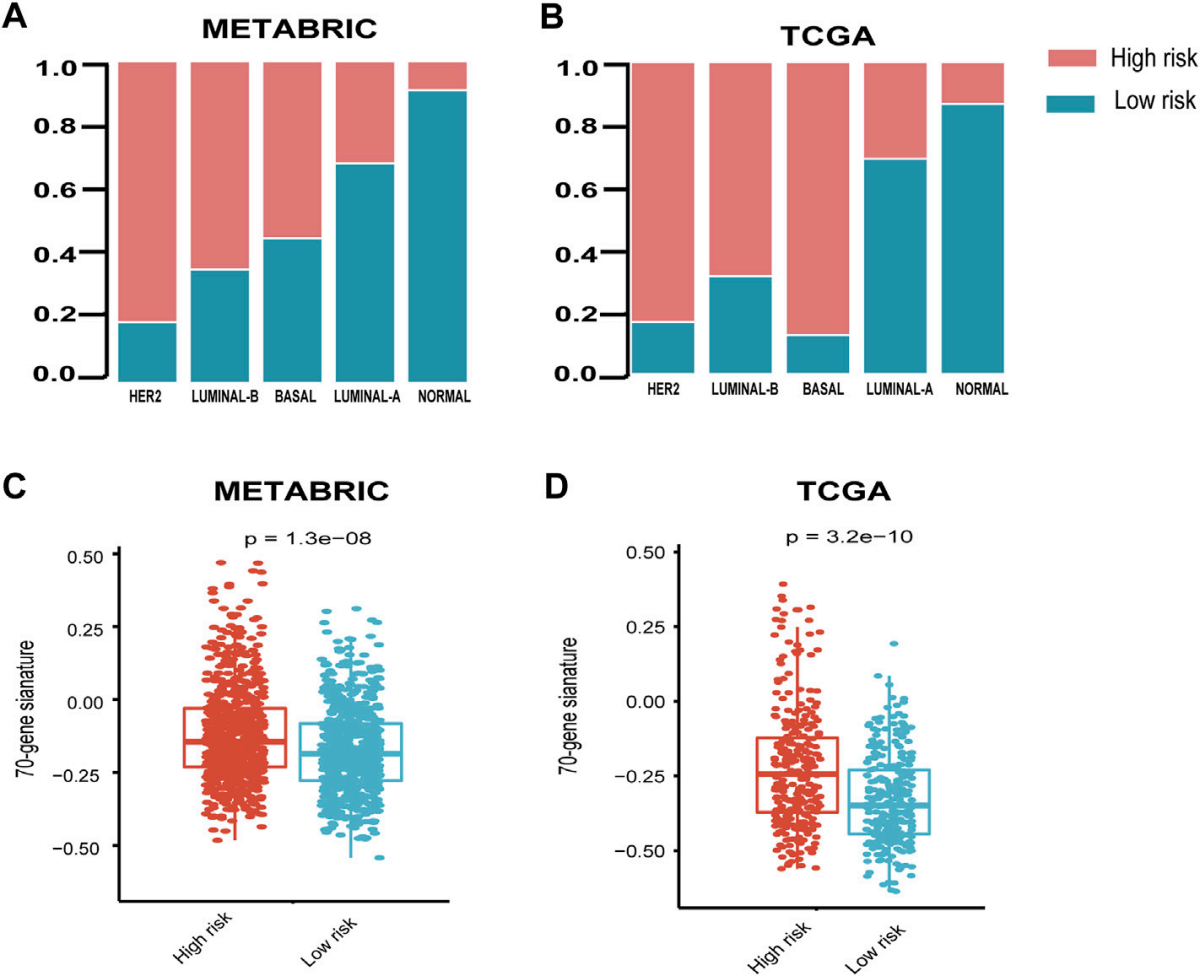
Relationship between seven-immune-related-gene IRS and the clinicopathological characteristics. **(A)** Relationship between IRS and PAM50 subtypes in the METABRIC cohort. **(B)** Relationship between IRS and PAM50 subtypes in TCGA cohort. **(C)** Relationship between IRS and 70-gene score in METABRIC and **(D)** TCGA cohorts. IRS: immune risk signature; METABRIC: Molecular Taxonomy of Breast Cancer International Consortium; TCGA: The Cancer Genome Atlas; ****: *p* < 0.0001. The Wilcoxon rank-sum test was performed.

It is shown that the high-risk group accounted for the highest percentage (>80%) in the disease with HER2-enriched subtype, followed by luminal-B and basal subtype in both METABRIC and TCGA cohorts (Figures 3A,B).

In order to explore the relationship between the published prognostic score and our risk signature, we calculated the 70-gene prognostic score using the genefu package. The results demonstrated that the 70-gene prognostic score was significantly higher in the high-risk group than the low-risk group (Figures 3C,D).

### Correlation of the Immune Risk Score With Tumor Microenvironment

**FIGURE 4:**
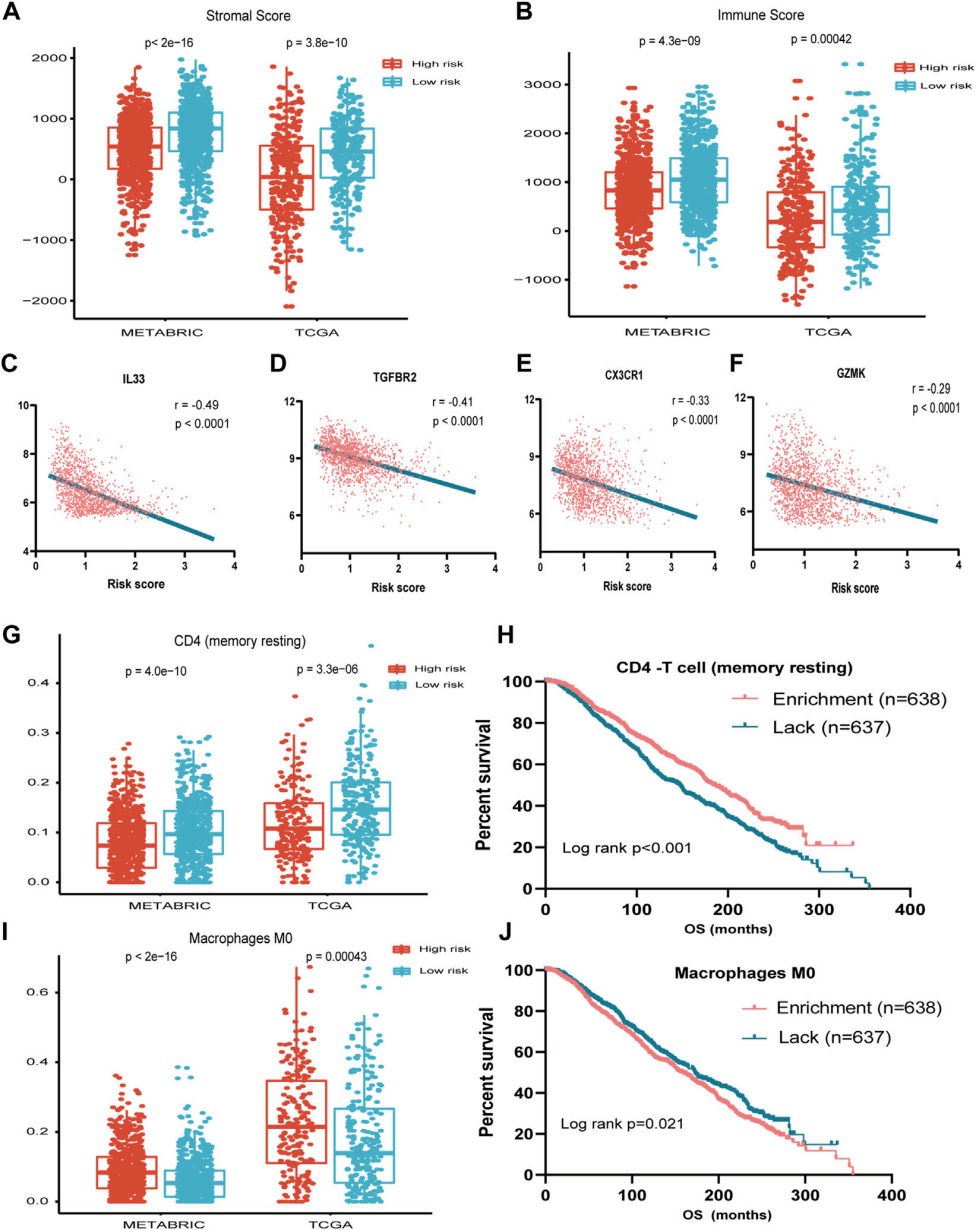
Association between seven-immune-related-gene IRS and TME. **(A)** Association between IRS and stromal score in METABRIC and TCGA cohorts. **(B)** Association between IRS and immune score in METABRIC and TCGA cohorts. **(C)** Association between IRS and IL33 in TCGA cohort. **(D)** Association between IRS and TGFBR2 in TCGA cohort. **(E)** Association between IRS and CX3CR1 in TCGA cohort. **(F)** Association between IRS and GZMK in TCGA cohort. **(G)** Association between IRS and resting memory CD4^+^ T cells in METABRIC and TCGA cohorts. **(H)** OS for patients with high or low resting memory CD4^+^ T cells in the METABRIC cohort. **(I)** Association between IRS and M0 cell score in METABRIC and TCGA cohorts. **(J)** OS for patients with high or low macrophage M0 cell score in the METABRIC cohort. IRS: immune risk signature; TME: tumor microenvironment; METABRIC: Molecular Taxonomy of Breast Cancer International Consortium; TCGA: The Cancer Genome Atlas; OS: overall survival; ***: *p* < 0.001; ****: *p* < 0.0001.

To address the correlation between tumor immunity and the risk signature, we applied the ESTIMATE and CIBERSORT algorithms to calculate the tumor purity and abundances of infiltrating stromal cells and immune cells in TCGA and METABRIC databases. As shown in Figures 4A,B, the stromal- and immune-scores were both significantly higher in the low-risk group. In addition, the risk signature was strongly anti-correlated with several important immune-related genes such as IL33 and TGFBR2 (Figures 4C–F).

By applying the CIBERSORT algorithm, the relative proportions of 22 immune cell subsets of ER- or PR-positive and HER2-negative BC in TCGA and METABRIC datasets were estimated. Consecutively, compared with the high-risk group, the level of CD4 memory resting T cell was increased, while the level of macrophage M0 was decreased significantly in the low-risk group (Figures 4G,I).

We further investigated the prognostic significance of the abundance of immune cells. The survival analysis showed that the high abundance of CD4 memory resting T cells was significantly associated with favorable prognosis (*p* < 0.001), while the high abundance of macrophage M0 was associated with unfavorable survival (*p* = 0.021) (Figures 4H–J).

### Immune Risk Score is Associated With Immune-and Cell Cycle-Related Phenotypes

**FIGURE 5:**
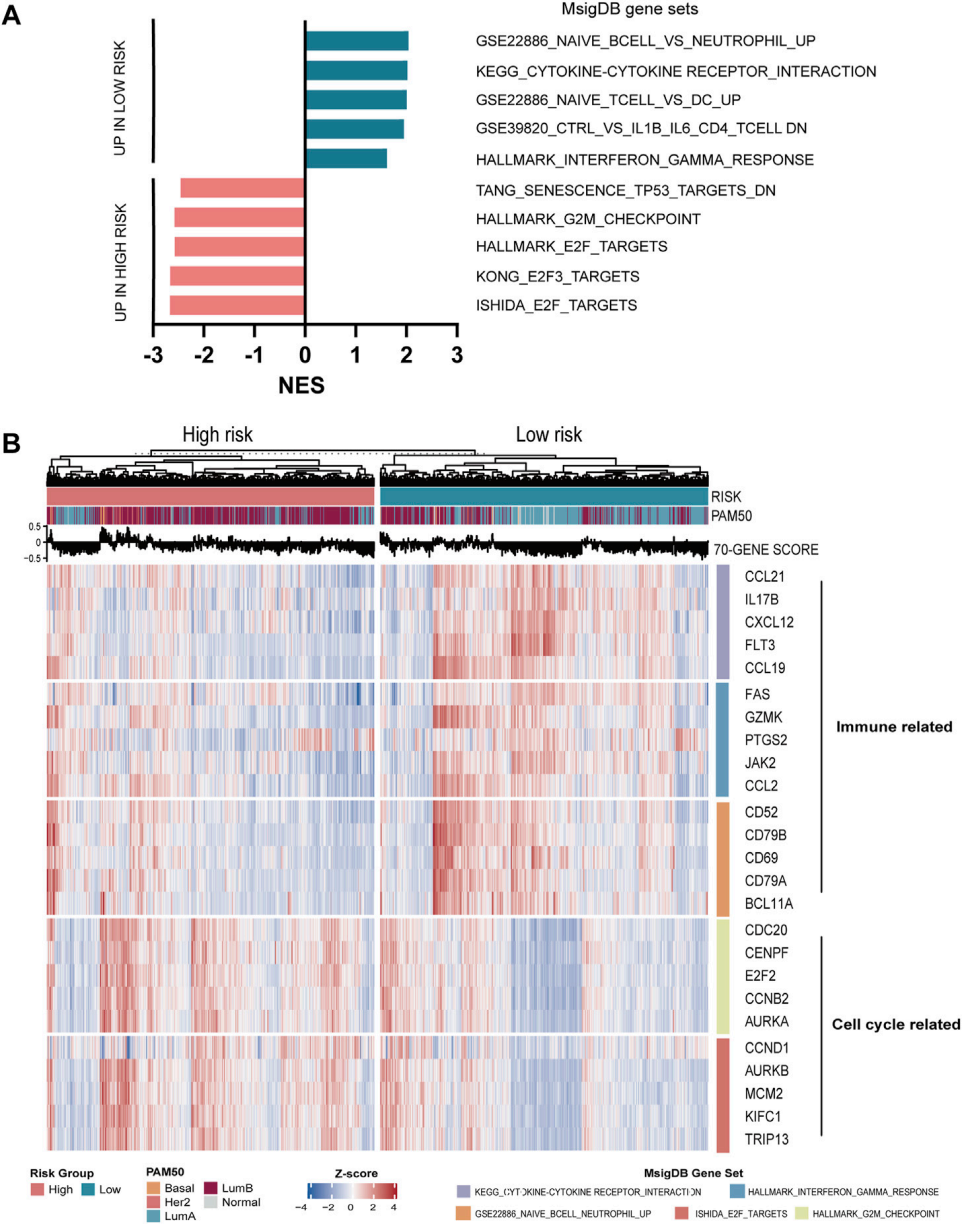
Functional analysis of seven-immune-related-gene IRS. **(A)** GSEA of IRS. **(B)** Relationship between seven-immune-related-gene IRS and signaling pathways. IRS: immune risk signature; GSEA: gene set enrichment analysis.

In order to further characterize the phenotype contributing to the worse prognosis in the high-risk group, we first performed differential expressed gene (DEG) analysis of the high-risk versus low-risk group in the METABRIC and TCGA datasets. Then, we performed GSEA using the collection of the MsigDB for these DEGs. As a result, we found that in the low-risk group, the immune-related pathway such as GSE22886_NAIVE_BCELL_VS._NEUTROPHIL_UP and KEGG_CYTOKINE–CYTOKINE RECEPTOR_INTERACTION were significantly enriched, while in the high-risk group, the cell cycle-related pathways such as KONG_E2F3_TARGETS and ISHIDA_E2F_TARGETS were enriched (Figure 5A). The heatmap displayed the expression of the core genes that contribute to pathway enrichment between the two groups. Notably, those cell cycle-related genes were predominantly upregulated in the high-risk group, while the immune-related genes were significantly upregulated in the low-risk group (Figure 5B).

**FIGURE 6:**
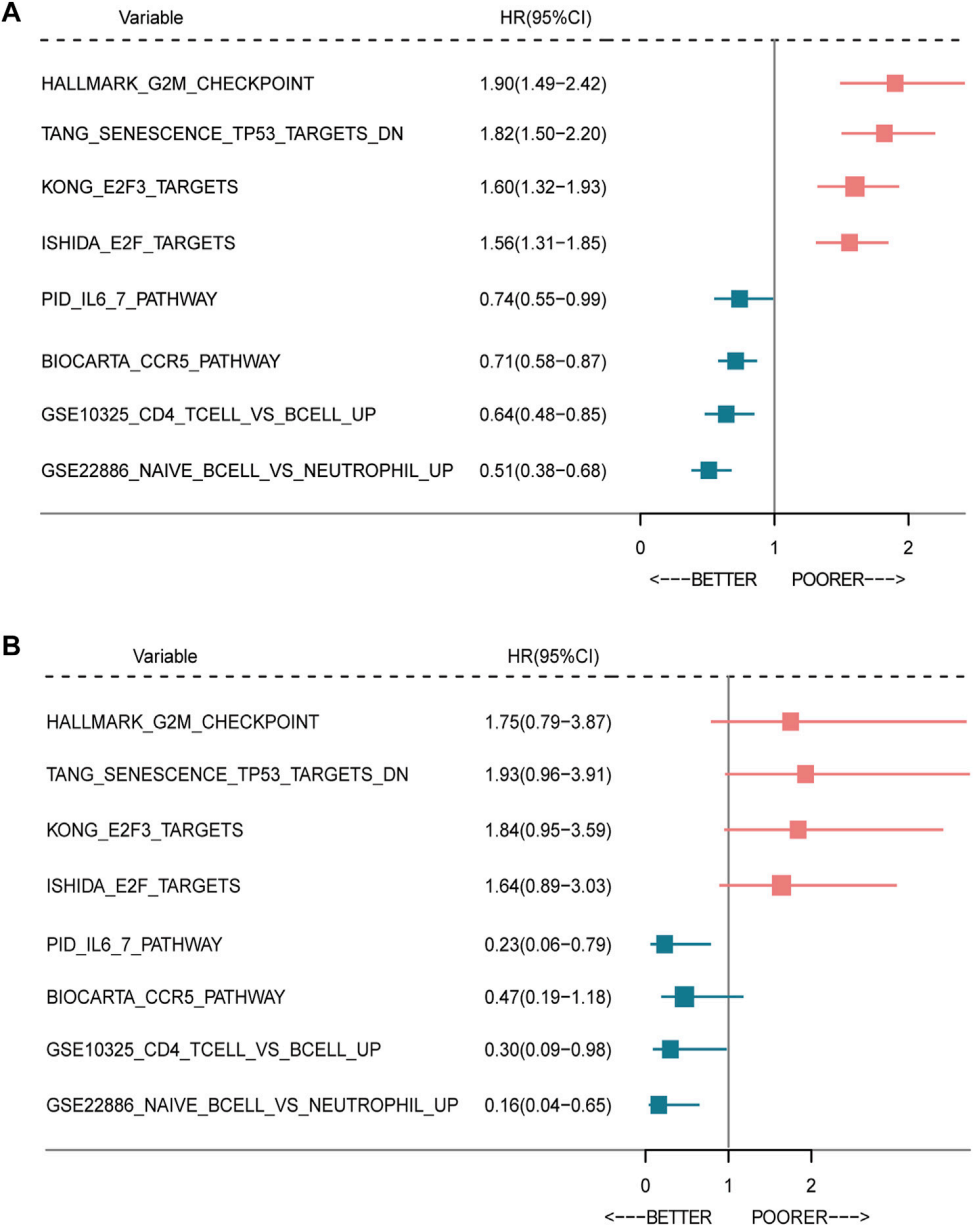
Forest plot of prognostic significance of the seven-immune-related-gene IRS. Prognostic factors in the METABRIC cohort **(A)** and TCGA cohort **(B)**. IRS: immune risk signature; BC: breast cancer; TCGA: The Cancer Genome Atlas.

In order to explore the relationship between the enrichment of pathway and prognosis, we selected those gene sets involving immune and cell cycle pathways to examine their prognostic significance, respectively. A total of 10 MsigDB gene sets were selected, and the gene set enrichment score of each sample was calculated by the GSVA method (Ritchie et al., 2015). The activity of the gene sets in each sample was estimated by the enrichment score. Then, the univariate Cox analysis was performed. As shown in Figures 6A,B, the enrichment of the cell cycle-related pathway was significantly associated with worse survival, and the enrichment of the immune-related pathway was significantly related with favorable survival in both METABRIC and TCGA databases.

## Discussion

ER (+) and/or PR (+) and HER2 (−) BC is a heterogeneous disease, which has a distinct profile of response to endocrine therapy. However, based on the immunohistochemical staining of ER, PR, and Ki67, the traditional classification does not fully distinguish heterogeneity in this group. Hence, it is crucial to determine a new classification method and to improve the prognosis.

The TME profiling has become a prediction model for BC classification and for selections of antitumor treatment. Emerging evidence has demonstrated that the TME plays an increasing role in screening tumor biomarkers, predicting the prognosis, and recently selecting patients for immunotherapy trials in BC (Xiao et al., 2019; Bonneau et al., 2020). Based on TCGA cohort, METABRIC cohort, and GSE21653 cohort, the article analyzed the characteristics of TME and its potential prognostic value in luminal BC. The results of risk stratification revealed that resting memory CD4^+^ T cells were significantly decreased in the high-risk group. Conversely, M0-type macrophages were remarkably increased in the high-risk group in both TCGA and METABRIC cohorts, which may have an effect on the prognosis.

In our study, two TME subtypes (high-risk group and low-risk group) were identified based on tumor immune cell compositions calculated by CIBERSORT and ESTIMATE algorithms. The results in our study demonstrated that the higher TMS risk was positively correlated with the resting memory CD4^+^ T cells and was negatively correlated with M0 macrophages, which also showed that enrichment of resting memory CD4^+^ T cells and lack of M0 macrophages had better prognoses in BC. Previous evidence has confirmed the prognostic values of TMS in various cancers (Netanely et al., 2016). One previous study has identified that the resting memory CD4^+^ T cells and M0 macrophages were positively correlated with the TMS score in colon adenocarcinoma (Chen and Zhao, 2021). In addition to higher TME scores, resting memory CD4^+^ T cells and M0 macrophages were usually associated with worse prognoses. Moreover, the following study showed that a high abundance of CD4^+^ memory T cells was associated with better survival in gastric cancer (Ning et al., 2020). Additionally, previous research confirmed that the high-risk group had higher M0 macrophages than the low-risk group in hepatocellular carcinoma (Long et al., 2019). Our findings added to the emerging body of evidence that the expression of immune genes could provide additional prognostic information.

In addition, we detected the functional enrichment pathways using GSEA in the high-risk and low-risk TME groups. These pathways were all unregulated in the high-risk TME group, including TANG_SENESCENCE_TP53_TARGETS_DN, HALLMARK_G2M_CHECKPOINT, and HALLMARK_E2F_TARGETS. Trabectedin, an anticancer drug targeting TME, exerted a potent antitumor activity against Hodgkin Reed–Sternberg by inducing the G2M cell cycle (Casagrande et al., 2021). Meanwhile, we identified the other immune- and cell cycle-related genes associated with BC, including CCL21, IL17B, CXCL12, FLT3, CCL19, GZMK, PTGS2, and JAK2. CCL21, a potential therapeutic target, enhanced the responsiveness to immune checkpoint blockade (Whyte et al., 2020).

Although this study analyzed the TME characteristics of BC in four cohorts with strict inclusive and exclusive criteria, it also has several limitations. First, the selection and recall bias of the study, which as a retrospective, are unavoidable. Second, the prognostic values could not be fully elucidated due to lacking of complete chemotherapy and radiotherapy regimens in the current study. Thus, the results should be interpreted with caution when establishing a correlation between IRS and specific treatment. Third, the detailed molecular mechanism has not been investigated in the current study. It is necessary to explore the underlying mechanisms behind the risk scores and poor survival outcomes of BC in further *in vitro* or *in vivo* experiments.

## Conclusion

In summary, we developed and validated a 7-gene prognostic signature based on immune gene expression in ER (+) and/or PR (+) and HER2 (−) BC, which displayed distinct patterns of prognosis and genomic features. If confirmed, these findings may have important clinical implications in risk stratification for precision oncology treatment in this population.

## Data Availability

Publicly available datasets were analyzed in this study. These data can be found at: https://www.ncbi.nlm.nih.gov/geo/query/acc.cgi?acc = GSE21653. Molecular Taxonomy of Breast Cancer International Consortium (METABRIC) and The Cancer Genome Atlas (TCGA) data were downloaded from the UCSC Cancer Browser website.

## Supplementary Material

Supplementary Figure S1 Model and selection of seven-immune-related-gene IRS. **(A, B)** Processes of seven immune-related gene selection by the LASSO Cox model. **(C)** Optimal number of clusters by the NbClust package. **(D, E)** Optimal number of clusters by the ConsensusClusterPlus package. IRS: immune risk signature.

Supplementary Table S1 List of 2,600 immune-related genes.
