# Supplementary figures and images for: Novel Immune-Related Gene Signature for Risk Stratification and Prognosis of Survival in ER (+) and/or PR (+) and HER2 (−) Breast Cancer
Source: Front Pharmacol. 2022 Jun 2;13:820437. doi: 10.3389/fphar.2022.820437 (PMC9201983; doi:10.3389/fphar.2022.820437)

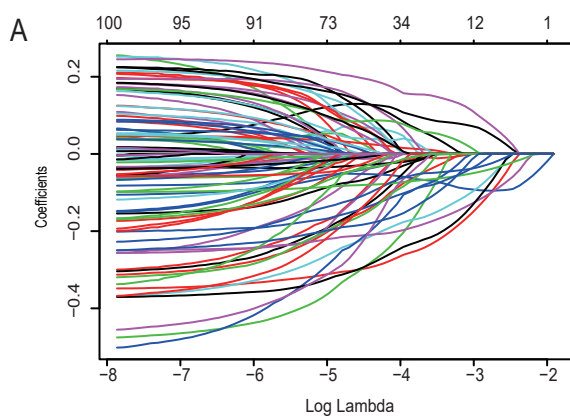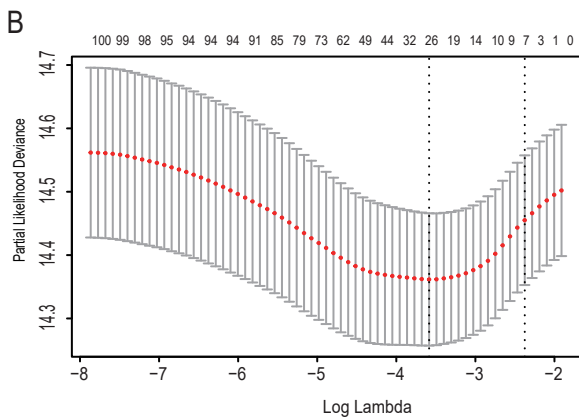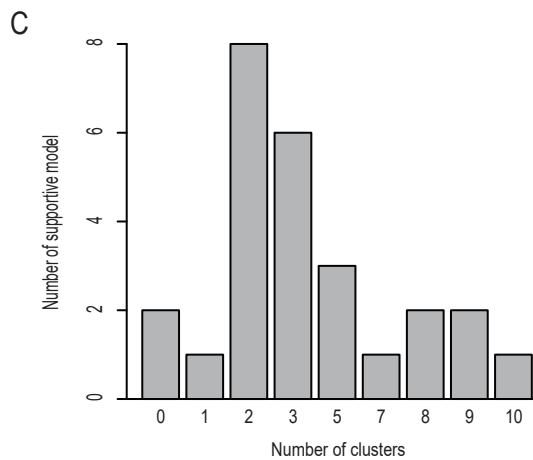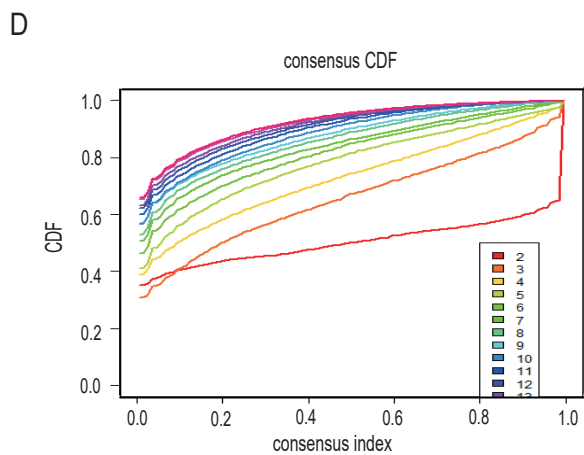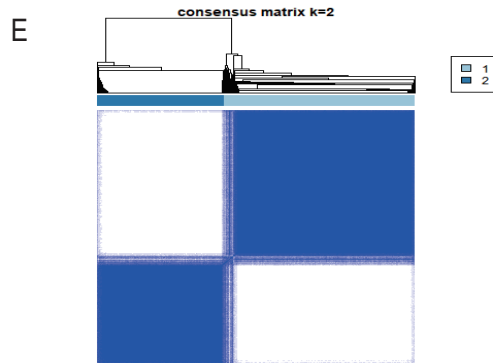

Supplement: Supplementary file 1 [file DataSheet1.PDF]
